# ABCA1/ApoE/HDL Signaling Pathway Facilitates Myelination and Oligodendrogenesis after Stroke

**DOI:** 10.3390/ijms21124369

**Published:** 2020-06-19

**Authors:** Li Li, Rongwen Li, Alex Zacharek, Fengjie Wang, Julie Landschoot-Ward, Michael Chopp, Jieli Chen, Xu Cui

**Affiliations:** 1 Department of Neurology, Henry Ford Hospital, Detroit, MI 48202, USA; lili@imm.ac.cn (L.L.); rli3@hfhs.org (R.L.); Azachar1@hfhs.org (A.Z.); fwang2@hfhs.org (F.W.); JLandsc@hfhs.org (J.L.-W.); mchopp1@hfhs.org (M.C.); JChen4@hfhs.org (J.C.); 2 Department of Physics, Oakland University, Rochester, MI 48309, USA

**Keywords:** ABCA1, ApoE, HDL, white matter, mylination, oligodendrogenesis, stroke

## Abstract

ATP-binding cassette transporter A1 (ABCA1) plays an important role in the regulation of apolipoprotein E (ApoE) and the biogenesis of high-density lipoprotein (HDL) cholesterol in the mammalian brain. Cholesterol is a major source for myelination. Here, we investigate whether ABCA1/ApoE/HDL contribute to myelin repair and oligodendrogenesis in the ischemic brain after stroke. Specific brain ABCA1-deficient (ABCA1^-B/-B^) and ABCA1-floxed (ABCA1^fl/fl^) control mice were subjected to permanent distal middle-cerebral-artery occlusion (dMCAo) and were intracerebrally administered (1) artificial mouse cerebrospinal fluid (CSF) as vehicle control, (2) human plasma HDL3, and (3) recombined human ApoE2 starting 24 h after dMCAo for 14 days. All stroke mice were sacrificed 21 days after dMCAo. The ABCA1^-B/-B^–dMCAo mice exhibit significantly reduced myelination and oligodendrogenesis in the ischemic brain as well as decreased functional outcome 21 days after stroke compared with ABCA1^fl/fl^ mice; administration of human ApoE2 or HDL3 in the ischemic brain significantly attenuates the deficits in myelination and oligodendrogenesis in ABCA1^-B/-B^–dMCAo mice ( *p* < 0.05, *n* = 9/group). In vitro, ABCA1^-B/-B^ reduces ApoE expression and decreases primary oligodendrocyte progenitor cell (OPC) migration and oligodendrocyte maturation; HDL3 and ApoE2 treatment significantly reverses ABCA1^-B/-B^-induced reduction in OPC migration and oligodendrocyte maturation. Our data indicate that the ABCA1/ApoE/HDL signaling pathway contributes to myelination and oligodendrogenesis in the ischemic brain after stroke.

## 1. Introduction

Stroke is a major cause of white matter (WM) damage, which induces long-term disability due to the limited axonal regeneration and remyelination in the inhibitory environment of the adult mammalian central nervous system (CNS) [1,2,3]. WM refers to areas of the CNS that are mainly composed of bundles of axons ensheathed with myelin, i.e., myelinated axons [4,5]. WM damage including axonal degeneration and the loss of myelin known as demyelination induces the disturbance of messages passing between different areas of gray matter within the CNS, which may occur even in the early stage of brain impairment and evokes serious neurological functional deficits after stroke [4,5,6]. WM-remodeling in the ischemic brain is essential for long-term stroke recovery [7]. 

Oligodendrocytes (OLs), the unique myelin-forming cells in the CNS, ensheath axons with myelin and maintain long-term axonal integrity. OL injury, for example stroke, causes a loss of myelin sheath and interruption of WM integrity and function [3]. Oligodendrogenesis is a regenerative process by which oligodendrocyte progenitor cells (OPCs) in the adult brain parenchyma stem cells [8,9,10] and primarily generated in the subventricular zone [11,12,13,14,15,16] multiply rapidly and proliferate and differentiate and mature into OLs [2]. Newly generated OLs within the peri-infarct WM myelinate sprouting axons (myelination) [8,17,18,19,20,21]. Therefore, oligodendrogenesis plays an important role in the WM remodeling during brain repair [22]. 

Cholesterol biosynthesis is a key pathway during myelination and disturbances are described in demyelinating diseases, such as multiple sclerosis [23]. The ATP-binding cassette transporter A1 (ABCA1), a major reverse cholesterol transporter, is a critical factor in the generation of apolipoprotein E (ApoE) and high-density lipoprotein (HDL) cholesterol. ApoE is the most abundant apolipoprotein in the brain and delivers lipids and cholesterols into cerebral cells [24,25,26,27]. Previous study demonstrated that the deletion of brain ABCA1 significantly reduces brain ApoE and HDL content [28,29], and it decreases myelin density and OLs and OPCs in the ischemic brain after stroke [30]. Brain ABCA1-deficient (ABCA1^-B/-B^) mice exhibit decreased WM remodeling as well as dysfunctional neurological outcome after stroke, and intracerebral supplementation of human ApoE or HDL reverses the deficits in ABCA1^-B/-B^ stroke mice [29,31]. However, the mechanisms underlying ABCA1-deficient induced deficits in WM remodeling after stroke are not fully understood. In this study, using ABCA1^-B/-B^ mice, we further investigate whether ABCA1, ApoE, and the HDL signaling pathway mediate axonal myelination and contribute to oligodendrogenesis and WM remodeling in the ischemic brain after stroke. 

## 2. Results

### 2.1. ABCA1^-B/-B^ Mice Exhibit Reduced Myelination in the WM Area

**Figure 1:**
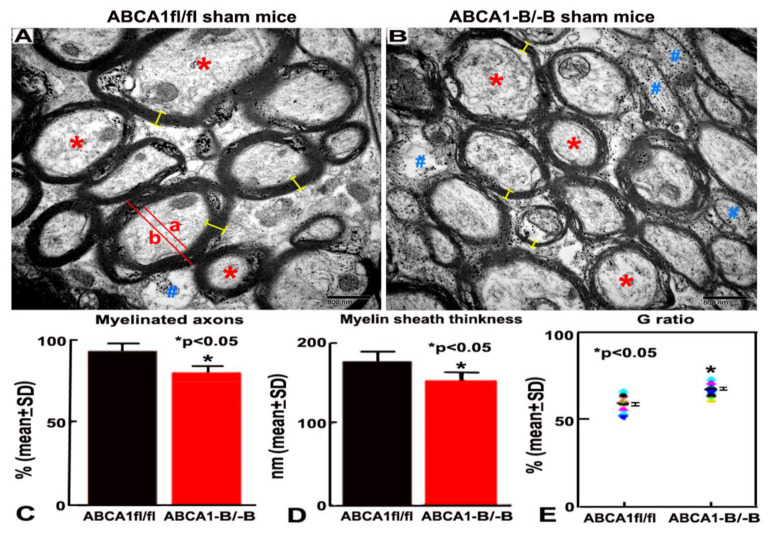
ATP-binding cassette transporter A1-deficient (ABCA1^-B/-B^) reduces myelination in the white matter (WM) of sham brain. (**A**,**B**) Electron microscopy (EM) images indicating the ultrastructure of WM in the corpus callosum (CC) from ABCA1-floxed (*ABCA1^fl/fl^*) (A) and *ABCA1**^-B/-B^*** (**B**) sham brain. (**C**–**E**) Quantitative data of percentage of myelinated axons, myelin sheath thickness, and G ratio. Stars (*) represent of myelinated axon characteristically on the outside of the myelin sheath of axons; Pond (**#**) represents hypomyelinated with reduplicated basal lamina or non-myelinated axon with thin myelin or complete lack of myelin around axons; Yellow bar (**-**) represents myelin thickness. G ratio = % of axon diameter (a)/diameter of axon with myelin sheath (b). Scale bar = 800 nm. * *p* < 0.05, *n* = 6/group.

To test whether brain ABCA1-deficient mice exhibit myelination deficit in the cerebral brain, the ultrastructure of the WM in the corpus callosum (CC) in the sham brain was analyzed in both ABCA1^-B/-B^ and ABCA1-floxed (ABCA1^fl/fl^) control mice by using image analysis of electron microscopy (EM). Compared with ABCA1^fl/fl^ mice (Figure 1A), ABCA1^-B/-B^ mice (Figure 1B) exhibit a decreased percentage of myelinated-axons (Figure 1C), decreased thickness of myelin sheath (Figure 1D), and an increased G ratio (Figure 1E) in the WM areas (*p* < 0.05, *n* = 6/group). These data indicate that the deletion of brain ABCA1 reduces myelination in the WM of brain.

### 2.2. ABCA1^-B/-B^ Stroke Mice did not Exhibit Change in the Ischemic Lesion Volume but Show Decreased Functional Outcome Compared with ABCA1^fl/f^ Stroke Mice; Administration of HDL3 or ApoE2 in ABCA1^-B/-B^ Stroke Mice Attenuated ABCA1^-B/-B^-Induced Functional Deficits 14 and 21 Days after Stroke

**Figure 2:**
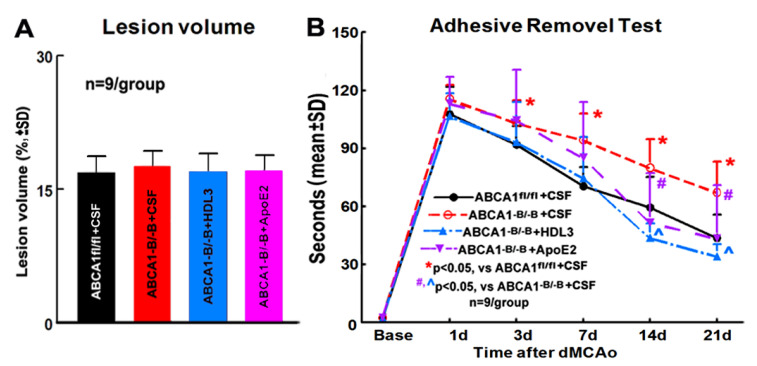
ABCA1^-B/-B^ stroke mice significantly decreases functional outcome compared to ABCA1^fl/fl^ stroke mice administered with cerebrospinal fluid (CSF); administration of high-density lipoprotein (HDL)3 or apolipoprotein E (ApoE)2 in ABCA1^-B/-B^ stroke mice advertises ABCA1^-B/-B^-induced functional deficit after stroke. (**A**) Quantitative data of lesion volume. (**B**) Quantitative data of the adhesive removal test. * *p* < 0.05, vs. *ABCA1^fl/fl^* stroke mice; #, ^ *p* < 0.05, vs. *ABCA1^-B/-B^* stroke mice; *n* = 9/group.

Assessment of infarct volume during the subacute stage post stroke overestimates true infarct volume because of edema [32]. Our previous study showed that ABCA1-deficient (ABCA1^-B/-B^) mice exhibited a marginal increase (*p* = 0.052) in lesion volume compared with ABCA1-floxed (ABCA1^fl/fl^) mice measured 7 days after stroke, with the lesion in ABCA1^-B/-B^ stroke mice likely incorporating more edema than the lesion in ABCA1^fl/fl^ stroke mice. However, in the present study, there was no lesion volume change observed between ABCA1^fl/fl^ and ABCA1^-B/-B^ stroke mice administered cerebrospinal fluid (CSF), and within ABCA1^-B/-B^ stroke mice administered CSF, HDL3, or ApoE2 21 days after stroke (Figure 2A, *n* = 9/group). However, compared with ABCA1^fl/fl^ stroke mice, the ABCA1^-B/-B^ stroke mice exhibit significantly decreased neurological functional outcome from 3 to 21 days after stroke. The administration of HDL3 or ApoE2 into the ischemic brain of ABCA1^-B/-B^ stroke mice starting 24 h and daily for 14 days significantly improve neurological functional outcome at 14 and 21 days after stroke (Figure 2B, *p* < 0.05, *n* = 9/group). These data show that the administration of HDL3 or ApoE2 into the ischemic brain of reverses ABCA1^-B/-B^ induced functional deficit after stroke. 

### 2.3. ABCA1^-B/-B^ Decreased Myelination in the CC of Ischemic Boundary Zone (IBZ) after Stroke; Administration of HDL3 or ApoE2 Attenuated the Deficits in the Myelination in the CC of IBZ in ABCA1^-B/-B^ Stroke Mice

**Figure 3:**
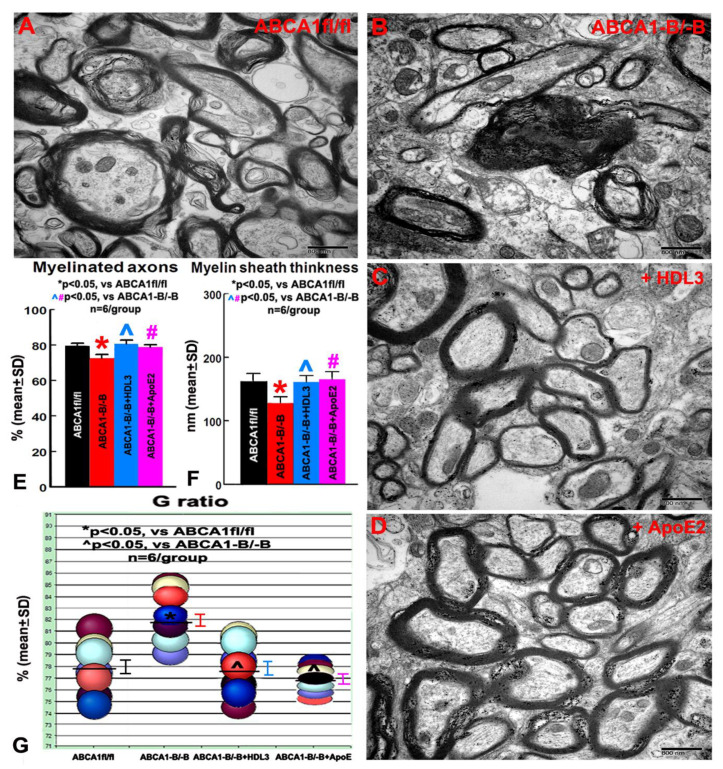
The electron microscopy (EM) images from the ischemic boundary zone (IBZ) of WM in ipsilateral ischemic brain show that ABCA1^-B/-B^ stroke mice exhibited decreased myelination compared with ABCA^fl/fl^ stroke mice. However, intracerebral administration of HDL3 or ApoE2 significantly increased myelination in the IBZ of WM in ABCA1^-B/-B^ stroke mice. (**A**) WM in the IBZ of ABCA^fl/fl^ stroke mice. (**B**) WM in the IBZ of ABCA^-B/-B^ stroke mice. (**C**) WM in the IBZ of ABCA^-B/-B^ stroke mice administered with HDL3. (**D**) WM in the IBZ of ABCA^-B/-B^ stroke mice administered with ApoE2. (**E**) Quantitative data of myelinated axons. (**F**) Quantitative data of the myelin sheath thickness. (**G**) Quantitative data of the G ratio. * *p* < 0.05, vs. ABCA1^fl/fl^ stroke mice; #, ^ *p* < 0.05, vs. ABCA1^-B/-B^ stroke mice. Scale bar = 800 nm, *n* = 6/group.

To investigate the mechanism underlying how brain ABCA1 deficiency decreases axonal myelination in the WM of the ischemic brain after stroke and how the administration of HDL3 and ApoE2 attenuate ABCA1^-B/-B^-induced reduction in myelination after stroke, the ultrastructural changes of WT in the CC of IBZ in the ipsilateral hemisphere were measured. The data show that the myelination in the CC of the IBZ significantly decreased in ABCA1^-B/-B^ stroke mice compared with ABCA1^fl/fl^ stroke mice 21 days after stroke. However, the intracerebral infusion of HDL3 or ApoE2 not only increases myelination in ABCA1^fl/fl^ stroke mice, it also significantly attenuates the reduction in the myelination in the CC of IBZ in ABCA1^-B/-B^ stroke mice 21 days after stroke (Figure 3A–C, *n* = 6/group). These data indicate that ABCA1 deficit decreases myelination in the IBZ of WM, while HDL3 or ApoE2 treatment attenuates ABCA1^-B/-B^-induced deficits in the myelination of WM, which may contribute to the neurological functional improvement after stroke. 

### 2.4. ABCA1^-B/-B^ Stroke Mice Exhibit Decreased Oligodendrogenesis Compared to ABCA1^fl/fl^ Stroke Mice; Administration of HDL3 or ApoE2 Significantly Attenuated the Reduced Oligodendrogenesis in the IBZ of ABCA1^-B/-B^ After Stroke

**Figure 4:**
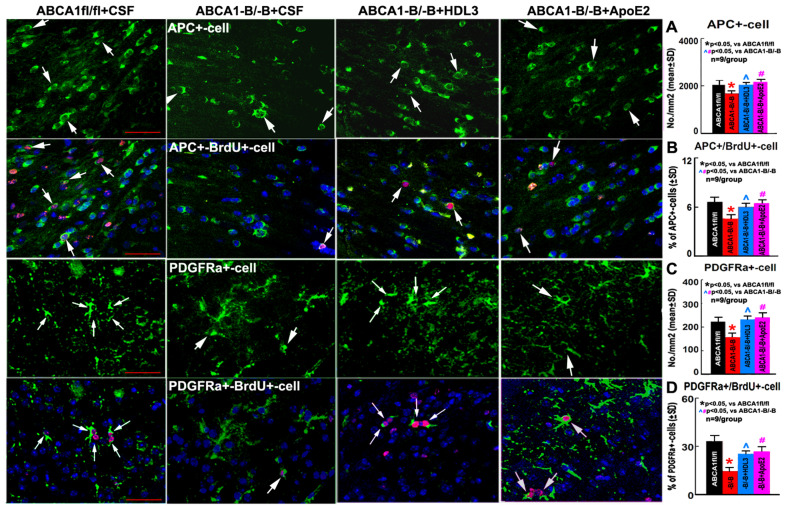
ABCA1^-B/-B^ vehicle stroke mice exhibit significantly increased oligodendrocyte (OL) loss and decreased oligodendrogenesis in the ischemic brain compared to ABCA1^fl/fl^ vehicle stroke mice; while treatment with HDL3 or ApoE2 reversed ABCA1^-B/-B^ induced the loss of OLs and oligodendrogenesis after stroke. (**A**) Adenomatous polyposis coli (APC^+^) cells and the quantitative data in the CC of IBZ in the ipsilateral brains of ABCA1^fl/fl^ and ABCA1^-B/-B^ stroke mice. (**B**) APC^+^/BrdU^+^-cells and the quantitative data in the CC of IBZ in the ipsilateral brains of ABCA1^fl/fl^ and ABCA1^-B/-B^ stroke mice. (**C**) Platelet-derived growth factor receptor α (PDGFRα^+^) cells and the quantitative data in the cortex of IBZ in the ipsilateral brain of ABCA1^fl/fl^ and ABCA1^-B/-B^ stroke mice. (**D**) PDGFRα^+^/BrdU^+^-cells and the quantitative data in the cortex of IBZ in the ipsilateral brain of ABCA1^fl/fl^ and ABCA1^-B/-B^ stroke mice. Scale bar = 50 µm; * *p* < 0.05, vs ABCA1^fl/fl^ stroke mice, ^, # *p* < 0.05, vs. ABCA1^-B/-B^ stroke mice. *n* = 9/group.

Our previous study demonstrated that ABCA1-deficent mice exhibit decreased numbers of OLs and OPCs in the ischemic brain 7 days after stroke [30]. In the present study, to further elucidate the cellular mechanism by which HDL3 or ApoE2 treatment increases OL number and oligodendrogenesis in the ischemic brain after stroke, the number of adenomatous polyposis coli (APC)-immunostaining positive cells (mature OLs, myelin-forming cells) in the CC and platelet-derived growth factor receptor α (PDGFRα)-immunostaining positive cells (a marker of OPCs) in the cortex of the IBZ as well as APC/PDGFRα double labeled with BrdU-immunostaining positive cells (newly differentiated OLs or newly born OPCs) in the ipsilateral brain of ABCA1^fl/fl^ and ABCA1^-B/-B^ vehicle control stroke mice, and ABCA1^-B/-B^ stroke treated with HDL3 or ApoE2 were measured. Consistent with our previous results [30], the present data show that the numbers of APC^+^-OLs and PDGFRα^+^-OPCs as well as APC^+^/BrdU^+^-OLs and PDGFRα^+^/BrdU^+^-OPCs in the IBZ of ipsilateral brain in ABCA1^-B/-B^ vehicle stroke mice significantly decreased compared with those in ABCA1^fl/fl^ vehicle stroke mice. However, compared with ABCA1^-B/-B^ vehicle stroke mice, the administration of HDL3 or ApoE2 into the ischemic brain significantly increased the numbers of APC^+^-OLs/PDGFRα^+^-OPCs and APC^+^/BrdU^+^-OLs/PDGFRα^+^/BrdU^+^-OPCs in the IBZ of ischemic brain in ABCA1^-B/-B^ mice 21 days after stroke (Figure 4, *p* < 0.05, *n* = 9/group). These data indicate that ABCA1 deficit induces an increased OL loss and decreased oligodendrogenesis, while the intracerebral infusion of HDL3 or ApoE2 significantly attenuates the OL loss and reduction of oligodendrogenesis in the ischemic brain after stroke. 

### 2.5. ABCA1^-B/-B^ Reduced ApoE and HDL Levels in OPCs 

**Figure 5:**
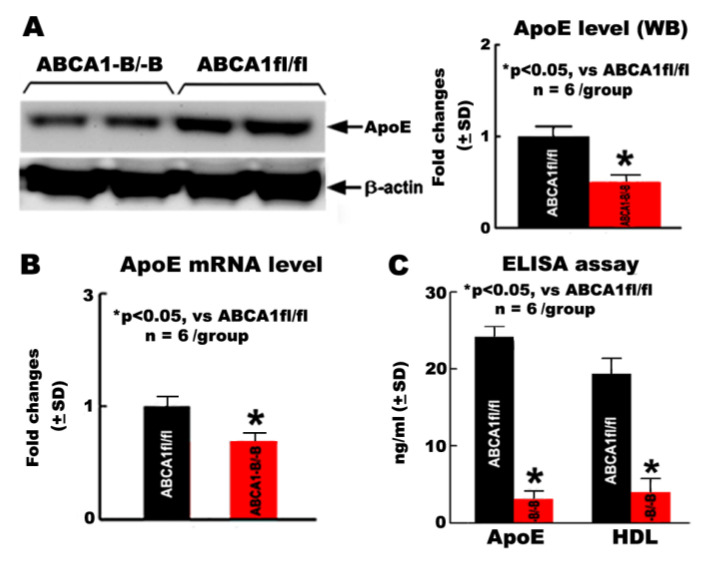
The ApoE and HDL level decreased in the primary cultured oligodendrocyte progenitor cells (OPCs) derived from ABCA1^-B/-B^ mice compared with OPCs derived from ABCA1^fl/fl^ mice. (**A**) ApoE protein level measurement by Western blot (WB) assay. (**B**) ApoE mRNA level measured by RT-PCR. (**C**) ApoE and HDL level measurement by ELISA. * *p* < 0.05, *n* = 6/group.

To investigate the mechanism underlying brain ABCA1 deficit-induced OL loss and oligodendrogenesis reduction, the in vitro primary OPC cultures isolated from the neonatal ABCA1^fl/fl^ or ABCA1^-B/-B^ mice were employed, respectively. The ApoE and HDL level in the OPCs were measured by using Western blot (WB) and real-time RT-PCR or ELISA assay. The data show that both level of ApoE protein/mRNA and HDL in the OPCs derived from ABCA1^-B/-B^ mice are significantly decreased compared with those in the OPCs derived from ABCA1^fl/fl^ mice (Figure 5, *p < 0.05, *n* = 6/group).

### 2.6. OPCs Derived from ABCA1^-B/-B^ Mice Exhibit Decreased Migration Compared with OPCs Derived from ABCA1^fl/fl^ Mice; HDL3 and ApoE2 Treatment Significantly Increase OPC Migration of OPCs Derived from ABCA1^-B/-B^ Mice 

**Figure 6:**
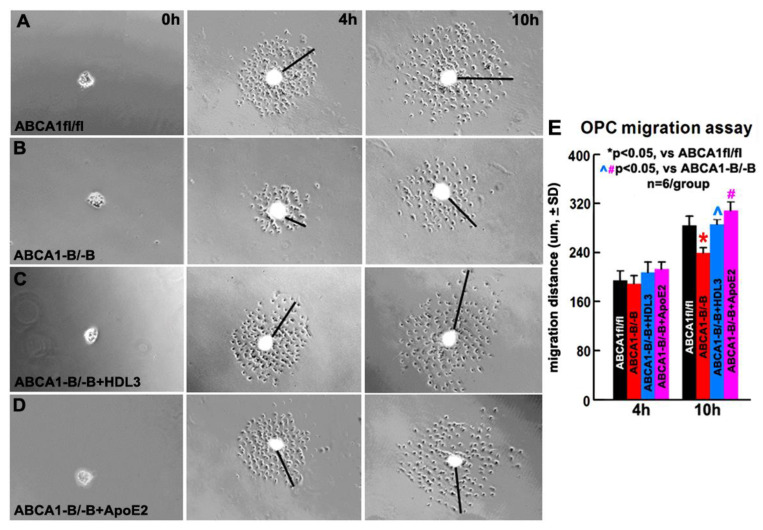
ABCA1^-B/-B^-OPCs exhibit decreased migration compared to ABCA1^fl/fl^-OPCs in vitro, while the treatment of ABCA1^-B/-B^-OPCs with HDL3 or ApoE2 reversed the reduction of migration. Images of OPC-sphere cultures in ABCA1^fl/fl^–OPCs (**A**), ABCA1^-B/-B^-OPCs (**B**), and ABCA1^-B/-B^-OPCs treated with HDL3 (**C**) or ApoE2 (**D**). The quantitative data of OPC migration distance (**E**) were acquired at 4 h or 10 h, respectively. * *p* < 0.05, vs. ABCA1^fl/fl^-OPCs; ^, # *p* < 0.05, vs. ABCA1^-B/-B^-OPCs; *n* = 6 wells/group.

In our previous in vitro study, the OPCs derived from ABCA1^-B/-B^ mice exhibit a decrease in proliferation and survival compared to OPC derived from ABCA1fl/fl mice [31]. Here, employing an OPC migration method specifically designed for use with mouse-derived cells [33], we further investigate whether ABCA1^-B/-B^ decreases OPC migration and if a supplement of ApoE/HDL attenuates ABCA1^-B/-B^-induced reduction in OPC migration. The data show that there is no significant difference in the migration distance between ABCA1^-B/-B^-OPCs and ABCA1^fl/fl^-OPCs measured at 4 h after culture. However, a significant decrease in the migration was observed in ABCA1^-B/-B^-OPCs 10 h after culture compared with ABCA1^fl/fl^-OPCs; treatment of ABCA1^-B/-B^-OPCs with ApoE2 or HDL3 significantly attenuated an ABCA1^-B/-B^-induced reduction in OPC migration 10 h after culture (Figure 6, *p* < 0.05, *n* = 6 wells/group). These data indicate that ABCA1^-B/-B^ decreases OPC migration, and supplemental HDL3 or ApoE2 attenuated ABCA1^-B/-B-^induced reduction in OPC migration.

### 2.7. ABCA1^-B/-B^ Reduced Maturation of OLs; HDL3 and ApoE2 Treatment Significantly Increase Maturation of OLs

**Figure 7:**
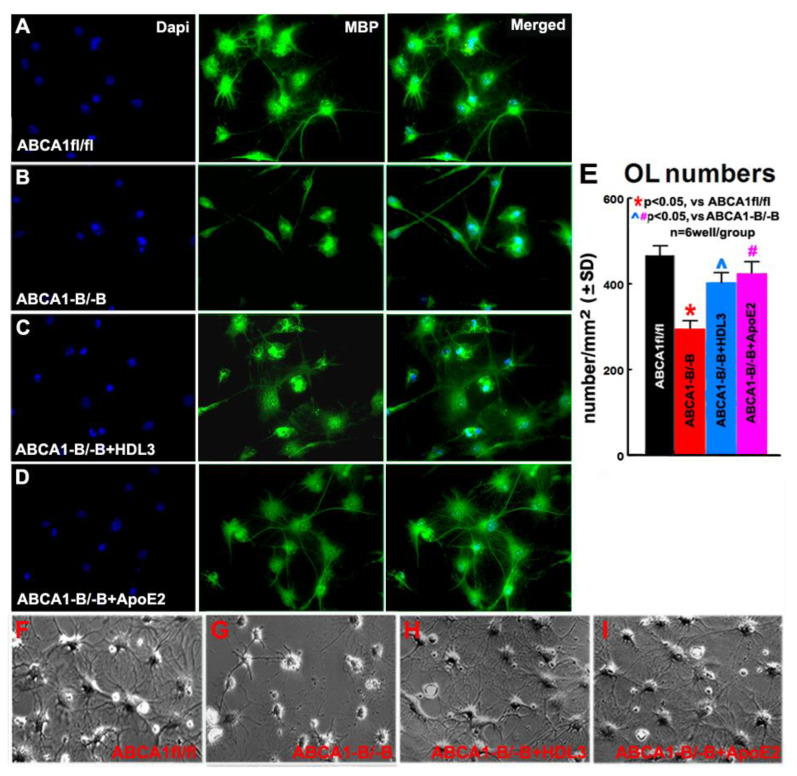
ABCA1-deficient decreases OL maturation, while treatment with ApoE2 or HDL3 significantly increases ABCA1^-B/-B^-derived OL maturation. The images of MBP^+^-OLs derived from ABCA1^fl/fl^ (**A**), ABCA1^-B/-B^ (**B**), treated ABCA1^-B/-B^-OPCs with HDL3 (**C**) or ApoE2 (**D**), and phase-contrast (**F**–**I**) obtained on DIV9, i.e., 6 days after initiation of treatment on DIV3. The quantities data of MBP+-OLs (**E**) were measured 6 days after treatment. * *p* < 0.05, vs. ABCA1^fl/fl^-OLs; **^, #** *p* < 0.05, vs. ABCA1^-B/-B^-OLs; *n* = 6 wells/group.

To further elucidate whether ABCA1^-B/-B^ affects OL maturation and whether ApoE/HDL mediates ABCA1^-B/-B^-induced reduction in OL maturation, the OL maturation assay was employed. Consistent with prior reports [32,34], at DIV9 cultured in the differentiation media, immunofluorescence microscopy images indicate most of cells are myelin basic protein (MBP, a marker of matured OLs [35]) positive OLs. The data show that the number of MBP^+^-OLs derived from ABCA1^-B/-B^ mice significantly decreased compared with MBP^+^-OLs derived from ABCA1^fl/fl^ mice. Concomitant with in vivo observation in myelination in the CC of IBZ, supplemented ApoE2 or HDL3 to ABCA1^-B/-B^-derived cells significantly increased MBP^+^-OL numbers (Figure 7, *p* < 0.05, *n* = 6/group) compared with non-treatment ABCA1^-B/-B^–derived cells. This morphological development is typical of the in vitro maturation of OLs. Using phase-contrast images to identify the shape and phenotype of cells, we observed that ABCA1^-B/-B^-derived cells exhibited fewer complex cell processes than ABCA1^fl/fl^-derived cells. However, HDL3 and ApoE2 treatment of ABCA1^-B/-B^-derived cells exhibit flattened and projected leaflet-like membrane structures (Figure 7). These data indicate that ABCA1^-B/-B^ decreases OPC maturation, and ApoE2/HDL3 treatment promotes ABCA1^-B/-B^-derived OPC differentiation into matured OLs. 

## 3. Discussion 

Thus far, the WM pathophysiology post-stroke remains relatively poorly understood compared to gray matter [2,36,37,38,39]. Possibly, a contributing factor to the absence of a clinically relevant neuroprotective agent for stroke may in part be attributed to the lack of emphasis on WM [2]. WM injury induced by stroke increases the risk of disability and poor prognosis of post-stroke rehabilitation [40]. Myelin, a lipid-rich membrane that surrounds axons, is critical for the propagation of nervous impulses and axonal maintenance. Stroke evokes demyelinating disorder in WM and remyelination limitation contributes to persistent disability [22,41]. However, the basic molecular mechanisms underlying demyelination and remyelination in response to ischemic stroke have not been fully clarified. EM is useful for the ultrastructure detection of myelination processes in animal models [42]. In the present study, in order to investigate the role of brain ABCA1 in WM injury after stroke, we initiated a study using EM to measure the ultra-structure of WM in the sham brain of ABCA1^-B/-B^ and ABCA1^fl/fl^ mice. We found that ABCA1^-B/-B^ mice exhibit significantly decreased numbers of myelinated axons and myelin sheath thickness, but increased G ratio in the sham brain compared with ABCA1^fl/fl^ mice. This finding is consistent with previous publications [28,43]. 

OLs are highly vulnerable to focal cerebral ischemia, and studies in animal models demonstrate that stroke results in loss of OLs, which contributes to the demyelination of myelin sheaths and impairs axonal function [8,16,44]. Remyelination occurs only from OPCs generated from the SVZ in adult rodent brain, where newly generated OPCs actively migrate to the injured WM and differentiate into mature OLs after brain injury [19,45,46]. Although most OPCs only proliferate without differentiation into OLs in the adult brain, a small quantity of OPCs provides a cellular reservoir for replacement of the lost OLs after injury [16,20,47]. The alteration of OLs, OPCs, and myelination were examined by using a rat MCAo model and found that the peri-infarct area exhibited a moderate reduction in the number of OLs and in myelin density, with a slight increase in OPCs at 2 days after MCAo. Subsequently, a steady increase in the number of OPCs and a gradual recovery of OLs were observed in the IBZ at 2 weeks after MCAo [48,49]. In this study, the ABCA1^-B/-B^ stroke mice exhibited a significantly reduced number of myelinated axons and myelin sheath thickness as well as reduced numbers of OLs in the IBZ of CC or cortex 21 days after distal middle-cerebral-artery occlusion (dMCAo), and induced severe functional deficits were observed from 3 to 21 days after stroke when compared with ABCA1^fl/fl^ stroke mice. These data suggest that ABCA1 plays a critical role in the myelination in brain WM, and the insufficient myelination in the brain of ABCA1^-B/-B^ mice may contribute to neurological deficits after stroke.

In addition to damage to OLs, disturbance of OPC proliferation, differentiation, and OL maturation results in deficient remyelination, which contributes to WM damage in the CNS [2,50]. Failure in remyelination can be attributed partially to an insufficient capacity of resident OPC to proliferate, migrate, differentiate, and initiate myelin membrane growth [46,51,52]. It has been suggested that increasing oligodendrogenesis promotes axonal myelination and neurological recovery in hypoxic/ischemic brains [53,54], whereas the transplantation of OPCs, and subsequent proliferation and differentiation to form mature myelinating OLs, could promote remyelination in stroke [7,55], multiple sclerosis [56], and spinal cord injury [7,57]. In animal models, some approaches, such as Nogo receptor blockage [46], Ferritin stimulation [58], mesenchymal stem cell transplantation [37,59,60], and trophic factor therapy (i.e., brain-derived neurotrophic factor-BDNF [61,62] or PDGFR [59]) have shown benefits in promoting remyelination and oligodendrogenesis. To further investigate whether ABCA1 deficit induces disturbance in oligodendrogenesis after stroke, we measured the number of newly born OPCs and newly matured OLs in the ischemic brain, and we found a reduced number of newly born OPCs and OLs in the IBZ of cortex and CC in ABCA1^-B/-B^ stroke mice compared with ABCA1^fl/fl^ stroke mice, which indicates that ABCA1^-B/-B^ reduces oligodendrogenesis after stroke. 

Cholesterol is rate-limiting for myelin biogenesis in the developing brain and in repair of the CNS, and it facilitates remyelination after brain WM damage [23]. Cholesterol synthesis and transportation in OLs are essential for optimal myelination and remyelination in pathological conditions such as multiple sclerosis [63,64]. A reduced synthesis of cholesterol by OLs results in impaired myelination [65]. ABCA1 plays a critical role in HDL cholesterol and ApoE synthesis and metabolism in the CNS [24,28,43,66,67,68,69]. Normally, plasma cholesterol is not taken up by the brain, and nearly all brain HDL and ApoE are synthesized in situ, primarily by astrocytes, thereby forming ApoE containing lipoprotein cholesterol particles, mainly via ABCA1 [24,66,70,71,72,73]. ABCA1^-B/-B^ mice have decreased brain levels of HDL [28,43] and ApoE, which is the most abundant cholesterol transporter [29,31]. In our previous studies using a model of primary cultured neurons derived from ABCA1^-B/-B^ mice, we showed that cholesterol reduction induces a significant decrease in neurite and axonal outgrowth. However, in the neuronal cultures, the reduced neuronal cholesterol level can be restored by supplementation of ApoE, which increases cholesterol uptake and redistribution via the ApoE2 receptor (ApoE2R) [31]. In the ABCA1^-B/-B^ mice, the brain levels of HDL and ApoE were also restored by intracerebral infusion of HDL3 or ApoE2 [29,31]. Previous studies have found that the intracerebral administration of human ApoE2 into ABCA1^fl/fl^ stroke mice significantly elevated the brain level of ApoE and HDL and increased gray/white matter density and neurogenesis, as well as improved neurological functional outcome after stroke [31]. To further investigate whether the ABCA1/ApoE/HDL signaling pathway facilitates axonal myelination and oligodendrogenesis after stroke, in the present study, ABCA1^–B/-B^ stroke mice were employed and treated with ApoE2 or HDL3. We demonstrate that the intracerebral administration of human HDL3 or ApoE2 in the ABCA1^-B/-B^ stroke mice remarkably promoted axonal myelination and augmented oligodendrogenesis in the IBZ of CC or cortex of ischemic brain as well as improved neurological functional outcome 21 days after stroke. Our data are consistent with others that show that using TRO19622, a small cholesterol-like compound, promotes myelin repair in a rat model of cuprizone-induced demyelination [74], and an ApoE mimetic COG112 stimulates axonal regeneration and remyelination after peripheral nerve injury [75]. Liver X receptors (LXRs) are nuclear oxysterol receptors that regulate genes involved in cholesterol homeostasis by targeting their genes of ABCA1 and ApoE, and therefore they play an important role in myelination [64]. The knock-out of LXRs in mice results in thinner myelin sheaths surrounding the axons [76]. The expression of LXR-beta and several established target genes (ABCA1, ApoE) was increased during OL differentiation, and treatment of primary neonatal rat OLs with synthetic LXR agonist T0901317 induced the expression of ABCA1 and ApoE, and it also resulted in an enhanced cholesterol efflux in the presence of HDL particles [64]. Our previous study shows that GW3965, an agonist of syntheses of LXRs, increases myelin density and promotes WM remodeling after stroke in ABCA1^fl/fl^ stroke mice via the upregulation of ABCA1 and by increasing the HDL level [29]. 

In normal newborn brain, the OPCs migrate along axonal tracts from the site of OPC generation and integrate, thereby forming WM. Similar migration and integration of grafted OPCs occurred in the spinal cord of normal myelinated rats and after a noninvasive grafting procedure [55]. In the current study, as we show in vitro, ABCA1^-B/-B^-derived OPCs have low levels of ApoE and decreased capability of migration and maturation to OLs, and that the administration of human ApoE2 or HDL3 reversed the deficits in OPC migration and maturation to OLs in ABCA1^-B/-B^-derived OPCs. These results suggest that ApoE2 and HDL3 treatment may restore the intracellular cholesterol imbalance in OPCs and OLs and leads to OL maturation, which may contribute to nerve fiber remyelination and axonal regeneration and enhance WM repair in the ischemic brain after stroke. 

Limitations: The present study is not an investigation of a stroke treatment; it is a proof-of-concept study that mainly focuses on mechanisms underlying the ABCA1/ApoE/HDL pathway in mediating myelination during stroke repair. Intracerebral infusion of human HDL3 or ApoE2 is not a clinically relevant approach. In the present study, we only investigate on male adult mice; we cannot exclude that female mice may have a different effect; in addition, age may be an impact factor that warrants investigation in future study. In the present study, the number of proliferating OPCs, i.e., PDGFRa+/BrdU+-OPCs was measured at 21 days after dMCAo. However, additional measurements of the baseline number of proliferating OPCs at 2 h or 1 day after BrdU injections are warranted. 

## 4. Materials and Methods

### 4.1. Experimental Groups and dMCAo Model 

For all in vivo studies, the use of animals and procedures was approved by the Institutional Animal Care and Use Committee of Henry Ford Health System (Code No. 1629, Approval Data 04/13, 2018), and they were performed in accordance with the Institutional Animal Care and Use Committee, National Institutes of Health and Animal Research: Reporting of *In Vivo* Experiments (ARRIVE) guidelines. In all animal groups, treatment and their identity were blinded to the surgeon and the investigators who performed behavior tests, lesion volume measurements, and EM and immunostaining analyses. 

ABCA1^-B/-B^ mice were originally generated by crossing loxP-flanked (floxed) ABCA1 mice with nestin-cre mice [28,77]. In the present study, the ABCA1^-B/-B^ mice and ABCA1^fl/fl^ mice were self-bred in the Bioresources of Henry Ford Health System. Male ABCA1^-B/-B^ mice were mated with ABCA1^fl/fl^ female mice. The litters are homologous and either ABCA1^-B/-B^ or ABCA1^fl/fl^, which were identified with genotyping assay [28,77]. Adult (6–7 months) male ABCA1^-B/-B^ (total 45 mice) and ABCA1^fl/fl^ (total 15 mice) control mice were subjected to permanent extraluminal right side dMCAo, which is a stroke model as previously described [31]. At 24 h after dMCAo, the ABCA1^-B/-B^ stroke mice were randomly divided into 3 groups (*n* = 15 each group) by a non-team member using the method of drawing different colored balls: 

(1) Vehicle-control group: mice were intraventricularly infused with artificial CSF (Tocris Bioscience, Minneapolis, MN, USA);

(2) HDL3 administration group: mice were intraventricularly infused with human plasma HDL3 (Cell Biolabs Inc., San Diego, CA, USA); 

(3) ApoE2 treatment group-mice were intraventricularly infused with recombined human ApoE2 (Sigma-Aldrich Corp., St. Louis, MO, USA). Briefly, HDL3 (25µg) or ApoE2 (25µg) was dissolved in 100 µL of artificial CSF in a micro-osmotic pump (D1002; Alzet, Cupertino, CA, USA), and then the micro pump was transplanted into the right lateral ventricle starting 24 h after dMCAo for 14 days. The dose selection is based on our previous publications [29,31]. 

The number of animals employed in vivo was determined a priori by power calculation; 9 animals per group provided 80% power at a significance level of < 0.05, assuming 20% difference in both mean and SD at the 95% confidence level and a two-sided test. Nine animals in each group were employed for the measurement of lesion volume, immunostaining, and functional outcome. All animals received BrdU injection (50 mg/kg, intraperitoneal injection), which was started 24 h after dMCAo and constantly once daily for a total of 7 days to label newborn cells. Six stroke and non-stroke animals in each group were used for the ultrastructure of axon and myelin analysis by EM imaging. All animals were sacrificed 21 days after dMCAo. 

### 4.2. Behavioal Testing

The behavioral testing, i.e., with adhesive removal test, was performed at 1, 3, 7, 14, and 21 days after dMCAo, and data were obtained by an individual who was blinded to mice treatment status. The test and the removal-time calculation method were performed as previously described [78]. 

### 4.3. Lesion Volume Measurement

All brains were fixed by transcardial perfusion with saline, followed by perfusion and immersion in 4% paraformaldehyde, and were then embedded in paraffin. Using a mouse brain matrix (Activational Systems Inc., Warren, MI, USA), the cerebral tissues were cut into seven equally spaced (1 mm) coronal blocks and a series of adjacent 6 µm thick sections were cut from each block. Seven sections were processed and stained with hematoxylin and eosin. For lesion volume measurement, the indirect lesion area was calculated, in which the intact area of the ipsilateral hemisphere was subtracted from the area of the contralateral hemisphere, which was calculated with a micro-computer imaging device (MCID) imaging analysis system (Imaging Research, ST. Catharines, ON, Canada) [79]. Lesion volume is presented as the volume percentage of the lesion compared with the contralateral hemisphere [80]. 

### 4.4. Quantification of Myelination on EM Images

For measurement of the ultrastructure of axonal myelination, 6 animals per group were transcardially perfused with saline followed by 4% paraformaldehyde and immersed in 2.5% glutaraldehyde buffer for 2 days. Briefly, 1 mm^2^ tissue blocks of the CC from the sham brains and the IBZ of ischemic brains were cut out and processed to ultrathin sections for EM analysis. Axonal structural change was identified at magnification of 7100× or 14,000× in a total of 6 areas per animal. The analysis of axonal myelination including the percentage of myelinated axons, the thickness of myelin sheath, and G ratio (axon diameter/axon wrapped with myelin diameter ×100%) were measured in 10 ultrathin sections, as previously described [81,82,83,84].

### 4.5. Immunohistostaining

For oligodendrogenesis measurement, antibodies used for the identification of OLs and OPCs were APC, a marker of mature OLs (Ab-1, OP44, 1:100; Calbiochem, San Diego, CA, USA) [85], and PDGFRα, a marker of OPCs (C-20, SC-338, 1:100; Chemicon, Temecula, CA, USA), respectively. To identify proliferating OLs and OPCs, double-immunostaining of APC/PDGFRα with BrdU and Dapi (Santa Cruz, Santa Cruz, CA, USA) were employed, as previously described [30]. Double immunolabeling was visualized by secondary antibodies conjugated to Fluorescein isothiocyanate and Cy3 (Jackson Immuno., West Grove, PA, USA). The total number of APC^+^/PDGFRα^+^ cells and APC^+^/PDGFRα^+^ colocalized with BrdU+ cells in the contralateral and the IBZ of cortex and CC in the ipsilateral were counted using an MCID imaging analysis system (Imaging Research, Toronto, ON, Canada) at 40× magnification images [86]. 

### 4.6. Preparation of OPCs

For the investigation of OPC migration and differentiation, in vitro primary culture OPC isolated from ABCA1^fl/fl^ or ABCA1^-B/-B^ mice were employed. For OPC culture, cells were dissociated from the cortex of neonatal mice post 2 to 3 days, as previous described [34,87]. At this age and tissue region, isolated OPCs generate more than 90% of the WM OLs in the CNS [88]. Briefly, the cells were maintained in a mixed glial culture medium in poly-d-lysine-coated 75-cm^2^ flasks at 37 °C/5% CO_2_ for 9 days. The flasks were shaken for 1 h on an orbital shaker (200 rpm) at 37 °C to remove microglia. Then, the OPCs were purified by overnight high-speed orbital shaking and then the suspension was collected (most of astrocytes and neurons adhering to the flask bottom were removed). Then, the purified OPC cells were cultured in 6-chamber slides coated with Poly-DL-Ornithine (10 μg/mL in PBS, P0421-100MG, Sigma-Aldrich, St. Louis, MO, USA) in OPC growth media: Dulbecco’s modified eagle medium (DMEM, Sigma-Aldrich, St. Louis, MO, USA) containing 1% GlutaMax (Life Technologies, New York, NY, USA), 1% penicillin/streptomycin, PDGF (10 ng/mL) and 1% transferrin and sodium selenite (ITS, Sigma-Aldrich, St. Louis, MO, USA) medium supplement. 

### 4.7. OPC Migration Measurement 

To further investigate the mechanism underlying ABCA1/ApoE/HDL signaling promotion of OPC migration, a new OPC migration method specifically suited for use with mouse-derived OPCs was employed, as previously described [33]. Briefly, the DIV3 purified OPCs were incubated in neurosphere growth medium (DMEM with 1% GlutaMAX, 1% ITS, 0.33% penicillin/streptomycin, 5 μg/mL insulin) [82], OPCs tend to form aggregates (henceforth termed the OPC sphere) and were suspended in the culture media [33]. At the fourth day after the formation of OPC spheres, similarly size of OPC spheres were selected and seeded individually onto poly-DL-ornithine-coated 6-chamber slides with one OPC sphere per well and changed to incubation in migration media following the previous description [33] with slight modification. The migration media consisted of DMEM supplemented with 1% GlutaMAX, 2% B27 (Gibco, New York, NY, USA), 100 µg/mL bovine serum albumin (BSA), 1% insulin, 1% ITS, 60 ng/mL progesterone, 400 ng/mL 3,3′,5-triiodo-L-thyronine, 400 ng/mL L-thyroxine, 16 µg/mL putrescine, 10 ng/µL PDGF-AA (PeproTech, Rocky Hill, NJ, USA), 50 ng/µL ciliary neurotrophic factor (CNTF; PeproTech, Rocky Hill, NJ, USA) and 1 µg/mL aphidicolin (Sigma-Aldrich, St. Louis, MO, USA). For the maintenance of OPCs in a precursor state, aphidicolin (mitotic inhibitor) was included in the migration media to control OPC proliferation. Cells were treated with or without HDL3 (40 ug/mL) or ApoE2 (4 ug/mL) for 10 h. The phase-contract images of OPCs were acquired at time zero to record their original diameter. Migration assays were fixed at 4 or 10 h. Using Photoshop, “exclusion zones” were digitally overlaid onto the time-zero residual OPC sphere, serving to define the starting point of migration; only cells beyond the exclusion zone were considered to have truly migrated. The average migration distance of 10 longest-migrated cells per well was quantified using MCID and averaged a total of 6 wells per group at 4 and 10 h, respectively. Triple independently repeated experiments were performed. 

### 4.8. OL Maturation Measurement 

For OL maturation measurement, the DIV3 purified OPCs derived from neonatal ABCA1^fl/f^ or ABCA1^−B/−B^ mice were cultured with differentiation media as previously described [34,87] and with little change. The differentiation media consist of DMEM containing 1% insulin, 1% ITS media supplement, 15 nM triiodothyronine, 10 ng/mL CNTF, and 1 × n-acetyl-l-cysteine (NAC; Sigma-Aldrich, A-8199, city, if any state, Country). On INV3, the cells were treated with or without HDL3 (40 ug/mL) or ApoE2 (4 ug/mL) for another 6 days. Anti-MBP antibody (1:1000, SMI-99, Covance, Indianapolis, IN, USA) immunostaining and Dapi were used to identify mature OLs [49]. The number of MBP+-OLs was calculated 6 days (DIV9) after treatment, as previously described [34,87]. For the identification of OL maturation, phase-contrast images were also acquired on DIV9.

### 4.9. RT-PCR

The OPC cultures derived from ABCA1^fl/fl^ and ABCA1^−B/−B^ were harvested. Total RNA was isolated, and quantitative PCR was performed in the ABI Prism 7000 sequence detection system, using the Quantitec SYBY Green PCR kit (Qiagen). The following primers were designed using Primer Express software (ABI). GAPDH: Fwd, AGAACATCATCCCTGCATCC; Rev: CACATTGGGGGTAGGAACAC. ApoE: Fwd, GAGGAACAGACCCAGCAAATAC; Rev: CAGAGGCCTGTATCTTCTCCAT. 

### 4.10. Western Blotting

Equal amounts of cell lysate were used for WB, and the following primary antibodies were used: anti-ApoE (1:1000, ab20874, Abcam, Cambridge, MA, USA) and anti-β-actin (1:10000; ab6276, Abcam, Cambridge, MA, USA).

### 4.11. Statistical Analysis

Data are presented as mean ± standard error (SD). Two-way ANOVA followed by Tukey’s post hoc test were performed for analysis lesion volume, functional outcome, EM measurement of remyelination and immunostaining, and in vitro OPC migration and differentiation. An independent *t* test was made for demyelination between ABCA1^fl/fl^ and ABCA1^−B/−B^ sham groups and ApoE/HDL level between ABCA1^fl/fl^-OPCs and ABCA1^−B/−B^–OPCs. A value of *p* < 0.05 was taken as significant.

## 5. Conclusions

Taken together, the present study suggests a therapeutic effect of the ABCA1/ApoE/HDL signaling pathway on WM remodeling in the ischemic brain after demyelination induced by stroke. Thus, the ABCA1/ApoE/HDL signaling pathway may provide a new clinical strategy for improving neurological functional recovery after stroke.
